# Activation of secondary cell wall biosynthesis by miR319‐targeted *TCP4* transcription factor

**DOI:** 10.1111/pbi.12715

**Published:** 2017-04-27

**Authors:** Xudong Sun, Chongde Wang, Nan Xiang, Xiong Li, Shihai Yang, JianCan Du, Yongping Yang, Yunqiang Yang

**Affiliations:** ^1^ Key Laboratory for Plant Diversity and Biogeography of East Asia Kunming Institute of Botany Chinese Academy of Sciences Kunming China; ^2^ Plant Germplasm and Genomics Center The Germplasm Bank of Wild Species Kunming Institute of Botany Chinese Academy of Sciences Kunming China; ^3^ Institute of Tibetan Plateau Research at Kunming Kunming Institute of Botany Chinese Academy of Sciences Kunming China; ^4^ College of Plant Protection Yunnan Agriculture University Kunming China

**Keywords:** miR319, *TCP4*, transcription factor, secondary cell wall biosynthesis, xylem vessel element differentiation

## Abstract

The overexpression of miR319 in plants results in delayed senescence, and high levels of miR319‐targeted *TCP4* transcription factor cause premature onset of this process. However, the underlying mechanisms of this pathway remain elusive. Here, we found that miR319 overexpression results in a decrease in *TCP4* abundance and secondary cell wall formation in the stem. Conversely, constitutive expression of miR319‐resistant *TCP4* promotes secondary cell wall formation, indicating that miR319‐mediated *TCP4* controls secondary cell wall formation during development. Further analysis revealed that TCP4 might directly bind the promoter of *VND7* to activate its expression, which triggers the expression of a *VND7* transcriptional network associated with secondary cell wall biosynthesis and programmed cell death and accelerates vessel formation. In addition, the development process gradually increased *TCP4* expression. These results suggest that miR319 and its target *TCP4* can act as switches that turn on secondary cell wall synthesis and programmed cell death.

## Introduction

Small RNAs, which are typically 20–24 nucleotides long, are important for gene and chromatin regulation in plants (Chen, 2009; Taylor *et al*., 2014). MicroRNAs (miRNAs), approximately 21 nucleotides in length, negatively regulate target genes by partly pairing to the corresponding mRNA and facilitating its cleavage. Some miRNAs are conserved in different species and are believed to facilitate evolutionarily conserved functions in regulating organogenesis (Chen, 2009). Usually, miRNAs are transcribed by RNA polymerase II into primary‐miRNAs (pri‐miRNAs) in the nucleus. The pri‐miRNAs are processed by microprocessor containing Drosha and dsRNA‐binding protein (DGCR8) to produce precursor‐miRNAs (pre‐miRNAs; Voinnet, 2009). In plants, DICER‐like 1 (DCL1) proteins cleave the pre‐miRNA. Unlike in animals, plant miRNAs are cleaved by DCL1 mainly in the nucleus rather than the cytoplasm, and then the cleaved duplex is translocated into the cytoplasm by HASTY. Once in the cytoplasm, the miRNAs are unwound into single mature miRNAs by a helicase, and the mature miRNAs finally enter the ribonucleoprotein complex known as the RNA‐induced silencing complex, where they regulate targeted gene expression (Chen, 2009; Jones‐Rhoades *et al*., 2006).

Several plant miRNAs are involved in plant growth, development and the stress response (Chen, 2009; Sunkar, 2010). The first described plant miRNA mutant *jaw*‐D was identified in the transgenic line overexpressing miR319. The major targets of miR319 are a series of *TCP* (*TEOSINTE BRANCHED1/CYCLOIDEA/PCF*) transcription factors, including *TCP2/3/4/10/24* (Palatnik *et al*., 2003). Several studies revealed that miR319 and its targets play multiple roles in plant developmental processes, such as leaf morphogenesis, jasmonic acid biosynthesis, senescence and flower development (Li *et al*., 2012; Nag *et al*., 2009; Palatnik *et al*., 2003; Schommer *et al*., 2008). In *Solanum lycopersicum* (tomato), the down‐regulation of several *TCPs* by ectopic expression of miR319 results in larger leaflets and continuous growth of the leaf margin, whereas reduced levels of miR319 or enhanced levels of *TCP* decrease leaf sizes (Ori *et al*., 2007).

Plant cells are enclosed in an extracellular matrix, the cell wall, that imparts structural support and regulate growth and differentiation. All plant cells have a thin primary cell wall and certain cell types such as sclerenchyma cells, also have a secondary wall layer, located between the primary wall and the plasma membrane. Secondary cell walls consist mainly of cellulose, hemicellulose and lignin (Zhong and Ye, 2007). The biosynthetic pathway associated with secondary cell wall formation is highly regulated at the transcriptional level. Several lines of evidence have demonstrated that a network of transcription factors regulates plant secondary wall biosynthesis (Zhong and Ye, 2007). In this network, the NAC (for NAM, ATAF1/2 and CUC2) domain transcription factors, including SND1, NST1, NST2, VND6 and VND7, are master switches that control a set of downstream functional factors, which in turn activate secondary cell wall biosynthesis factors, such as *SND2*,* SND3*,* MYB20*,* MYB102* and *KNAT7*. This activation initiates the expression of secondary cell wall biosynthesis genes, leading to a massive deposition of the secondary wall in cells (Ellis *et al*., 2014; Zhong *et al*., 2006).

VND7 belongs to the NAC transcription factor family and is preferentially expressed in differentiating xylem vessel elements (Kubo *et al*., 2005; Yamaguchi *et al*., 2010b). *VND7* overexpression can induce the ectopic differentiation of protoxylem‐like vessels and result in a pale colour and death (Kubo *et al*., 2005; Yamaguchi *et al*., 2010b). Functional suppression of *VND7* causes defects in the formation of vessel elements. A broad range of putative direct target genes of VND7 has been identified through transcriptome analysis and encodes transcription factors, irregular xylem proteins and proteolytic enzymes (Yamaguchi *et al*., 2011). These results strongly suggest that *VND7* acts as a key regulator of xylem vessel differentiation.

A previous study showed that miR319 controls jasmonate biosynthesis and senescence through miR319‐targeted *TCP4*, which can bind to the promoter of the *LOX2* gene that is responsible for jasmonic acid biosynthesis (Schommer *et al*., 2008). However, the pathways that regulate senescence remain unclear. In the present study, we show that *TCP4* associates with the promoter region of *VND7* to directly activate its expression, which regulates the differentiation of all types of xylem vessels in roots and shoots (Yamaguchi *et al*., 2008). The disruption of *TCP4* resulted in the decreased formation of the secondary cell wall and the decreased differentiation of xylem vessel elements. Furthermore, we found that gradually increasing the level of *TCP4* corresponded to a gradual increase in the levels of *VND7* transcripts in the development processes. These data suggest that *TCP4* may be involved in xylem vessel differentiation via activating *VND7* transcription.

## Results

### Generation of stable transgenic lines overexpressing *rTCP4*

**Figure 1:**
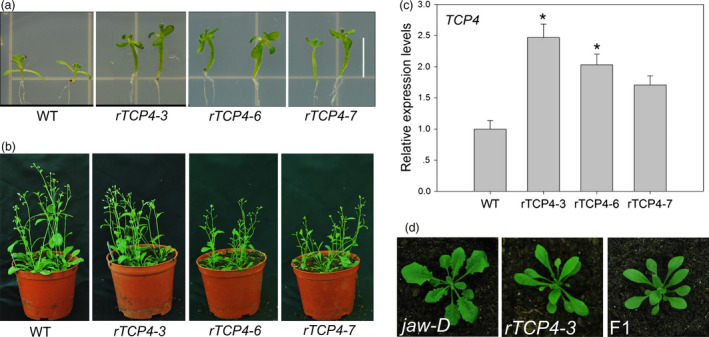
Phenotypes of rTCP4 transgenic plants. (a) Wild‐type and *rTCP4* 2‐week‐old seedlings. (b) Wild‐type and *rTCP4* 6‐week‐old seedlings. (c) Relative expression of *TCP4* in transgenic plants. Transcript levels were normalized to UBQ10 and then expressed relative to Col‐0. The expression level of *TCP4* in the wild type is set to 1. Error bars represent SE of triplicate experiments. (*P < 0.01). (d) Rosettes of 25‐day‐old seedlings. F1, the progeny between *rTCP4‐3* and *jaw‐*D. Bar = 0.5 cm.

Plants overexpressing miR319‐resistant *TCP4* (*rTCP4:GFP*) have a shorter life span than wild‐type (WT) plants and do not produce seeds (Schommer *et al*., 2008). However, three independent transgenic lines with weaker phenotypes, meaning that they produced seeds, were selected in this study. Three lines had long hypocotyls, epinastic cotyledons and smaller rosette leaves; further, their aerial parts were darker green than those of the wild type (Figure 1a), similar to the overexpression of *rTCP4:GFP* phenotype (Schommer *et al*., 2008). All three lines exhibited a normal life cycle (Figure 1b). Quantitative real‐time polymerase chain reaction (qRT‐PCR) analysis confirmed the up‐regulation of *TCP4* levels (Figure 1c). The *rTCP4*‐*3* transgenic plants showed the highest *TCP4* expression level, almost 2.5‐fold higher than WT, while *rTCP4‐6* plants showed a twofold increase, and *rTCP4‐7* plants showed a 1.7‐fold increase. Notably, even the *rTCP4*‐*7* transgenic plants with slight up‐regulation of *TCP4* exhibited the *rTCP4*‐*GFP* phenotype (Figure 1a). *Jaw‐D*,* a miR319a* overexpressing line, has crinkling leaves with cutting margins (Palatnik *et al*., 2003). When the *rTCP4‐3* line was crossed with *jaw*‐D plants, the phenotype of the heterozygote F1 plants alleviated the phenotype of the *jaw*‐D plants (Figure 1d). The fact that slight up‐regulation of *TCP4* affects plant growth and development indicates that the TCP4 plays a vital role in these processes.

### MiR319‐targeted *TCP4* is involved in xylem vessel element formation

**Figure 2:**
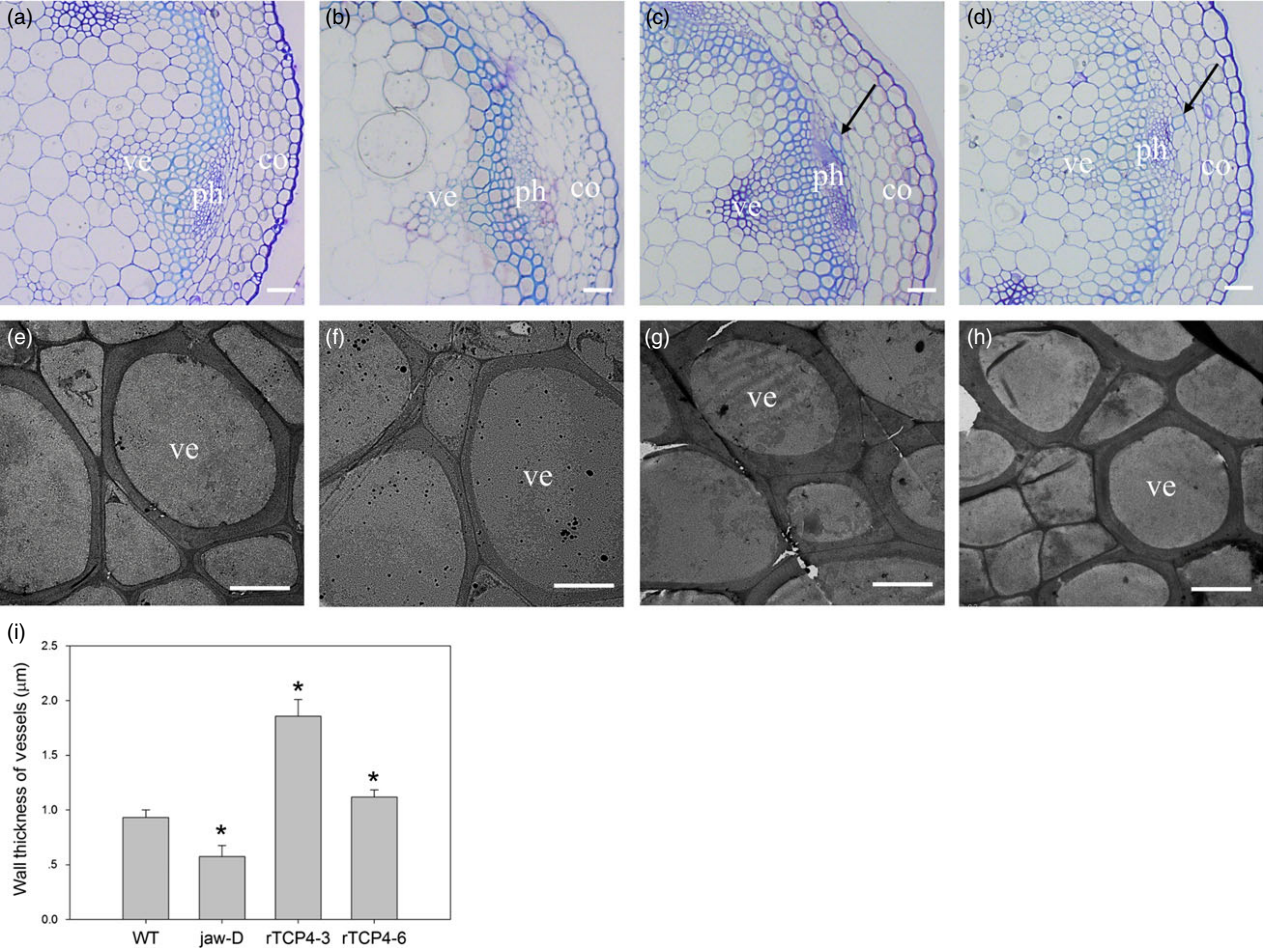
Cross‐sections of the stem in 6‐week‐old plants. (a–d) Cross‐sections of vascular bundles in the 15‐cm‐high stems of 6‐week‐old plants. Note that black arrow indicates increased vessel element formation. (e–h) Transmission electron micrographs of vessel walls in the stems of 6‐week‐old plants. (a, e) Wild type. (b, f) *jaw*‐D. (c, g) *rTCP4*‐*3*. (d, h) *rTCP4*‐*6*. co, cortex; ph, phloem; ve, vessel. (i) Wall thickness of the vessel elements in the stems of wild‐type, *jaw*‐D and transgenic plants. The wall thickness was measured from transmission electron micrographs. The differences in wall thickness of the xylem vessel elements in the stem between the wild type and the other lines are statistically significant (*P < 0.05). The data are the means (μm) of 60 cells, which are from three plants of each genotype. Bars = 20 μm (a–d), 5 μm (e–h).

When we transferred the transgenic plants to soil, we found that the hypocotyls of the *rTCP4*‐*3*,* rTCP4*‐*6* and *rTCP4*‐*7* transgenic plants were less flexible than WT plants. Thus, we speculated that lignification might be increased in the transformants. To examine this hypothesis, we selected *rTCP4*‐*3* and *rTCP4*‐*6* transgenic plants for further investigation. Histological studies were performed to investigate the differences in secondary wall structure among the WT, *jaw*‐D plants and *rTCP4*‐*OX* lines. Cross‐sections of the basal parts of the stems revealed that xylem vessel elements were more frequent in stems of *rTCP4*‐*OX* lines (Figure 2c,d). Transmission electron micrographs showed that the wall thickness of vessel elements in *jaw*‐D plants was clearly reduced compared with WT plants (Figure 2f,i). The overexpression of *rTCP4* resulted in significantly increased wall thickness of the vessels (Figure 2g,h,i), and the magnitude of the effect correlated with the expression level of *TCP4* (Figure 1c). These results indicate that TCP4 plays a vital role in vessel formation.

### Overexpression of *TCP4* causes increased deposition of lignin and cellulose

**Figure 3:**
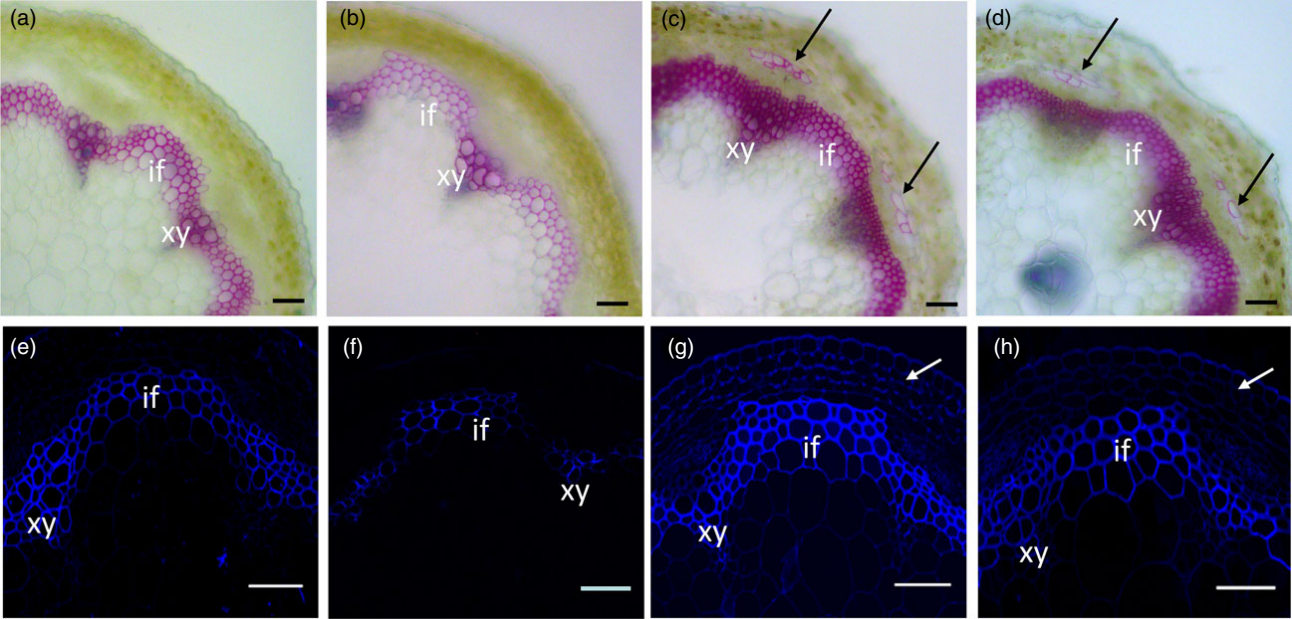
Modulation of lignin and secondary cell wall cellulose by miR319 and *TCP4*. Stem sections were stained with phloroglucinol‐HCl or Calcofluor White to detect lignin or secondary wall cellulose, respectively. (a‐d) Phloroglucinol‐HCl staining (red colour) revealed weaker lignin signals within the interfascicular fibres and xylem bundles in the *jaw*‐D plants (b) compared with the control wild‐type plants (a). The strongest signals were observed in the *rTCP4*‐*3* (c) and *rTCP4*‐*6* (d) lines. Note that increased lignin deposition was detected in the stem of *rTCP4*‐*3* (c) and *rTCP4*‐*6* (d). (e–h) Calcofluor White staining (blue colour) of stem sections showing cellulose staining in the walls of interfascicular fibres and xylem cells in seedling stems of the wild type (e), *jaw*‐D (f), and *rTCP4*‐*3* (g) and *rTCP4*‐*6* (h) lines. Note that strong cellulose signals were detected in the stem cortex of *rTCP4*‐*3* (g) and *rTCP4*‐*6* (h). xy, xylem; if, interfascicular fibre. Bars = 50 μm.

Secondary cell walls are mainly composed of lignin, cellulose and hemicellulose. To further investigate the function of *TCP4* in the regulation of secondary wall biosynthesis, we examined whether the observed increase in cell wall thickness corresponded to an increased deposition of lignin, cellulose or both. As shown in Figure 3, conventional Wiesner histochemical stains (phloroglucinol‐HCl, in which a violet‐red colour is indicative of lignins) exhibited less intense coloration in the secondary cell walls of *jaw*‐D plants than in the controls, but higher intensity in the *rTCP4‐OX* lines (Figure 3a–d). Ectopic lignin staining was also observed in the stem cortex of *rTCP4‐OX* lines (Figure 3c,d). These data indicate a reduction in lignin content in the secondary cell wall of *jaw*‐D plants and increased lignin content in the *rTCP4‐OX* lines.

Similarly, histological staining of cellulose in the stems (using Calcofluor White, in which a blue colour is indicative of secondary wall cellulose), under epifluorescence microscopy, revealed weaker fluorescence within the interfascicular fibres and xylem bundles in the *jaw*‐D plants compared with the control WT plants (Figure 3e,f). However, the fluorescence intensity was stronger in the *rTCP4*‐*OX* lines (Figure 3g,h). These results indicate that TCP4 is involved in secondary wall biosynthesis affecting the abundance of both cellulose and lignin.

### TCP4 might directly activate the expression of *VND7* for xylem vessel element formation

**Figure 4:**
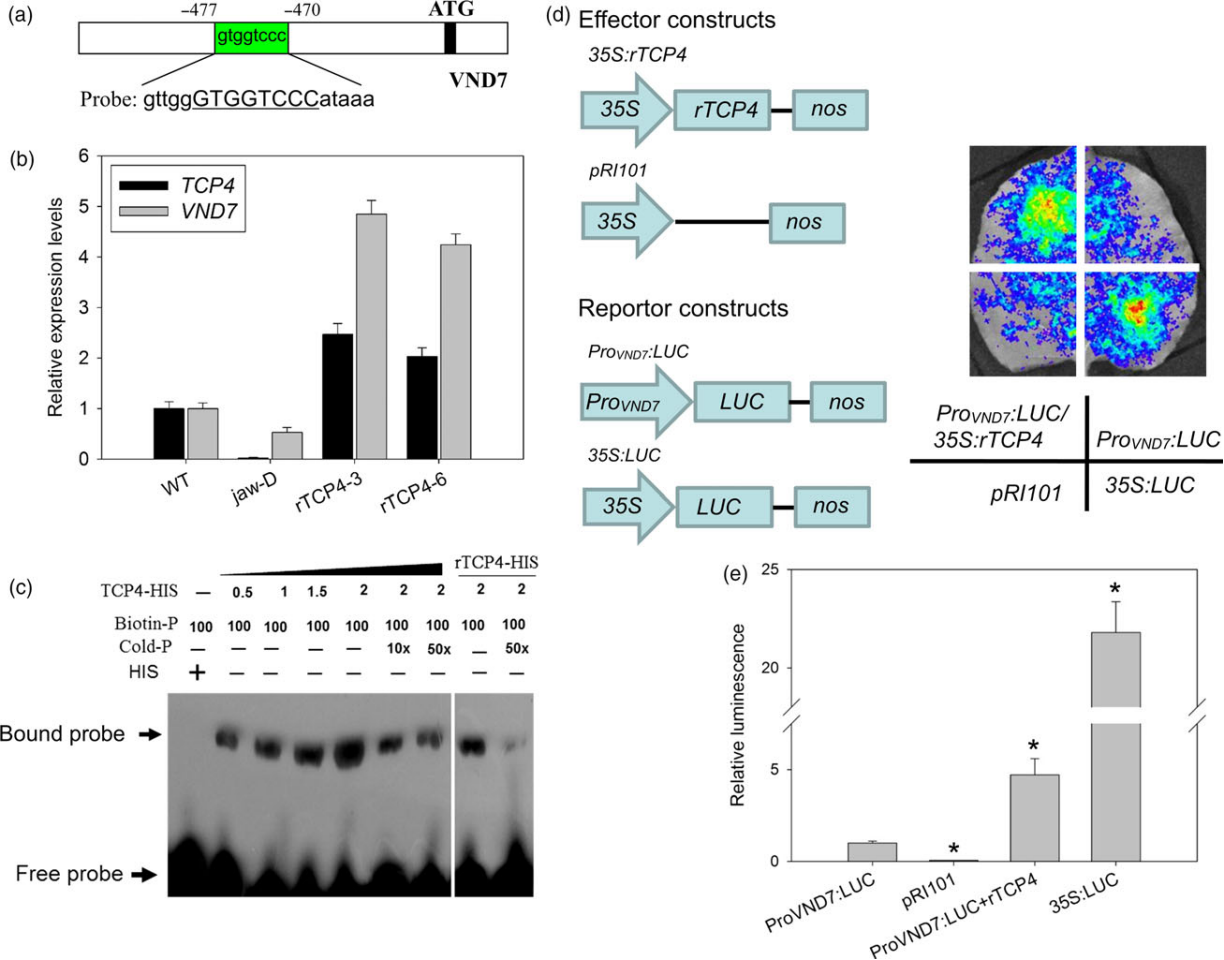
TCP4 binds to the promoter region of *VND7*. (a) The promoter sequence of the *VND7* gene. The green box represents the putative TCP4‐binding element (GTGGTCCC). (b) qRT‐PCR analysis of *TCP4* and *VND7* expression in Col‐0, *jaw*‐D plants and *rTCP4*‐*OX* lines. The expression levels of *TCP4* and *VND7* in the wild type are set to 1, respectively. Relative expression of each gene was calculated by normalizing to the value in WT plants. Error bars represent SE of triplicate experiments. (c) *In vitro* EMSA assay showing that the TCP4‐HIS fusion protein, but not the negative control 6HIS, binds to the *VND7* promoter (Probe). rTCP4‐HIS also bounds to the promoter region of the *VND7*. Biotin‐P indicates the biotin‐labelled probe, and Cold‐P indicates the probe without biotin labelling. Competition for TCP4 binding was performed with 10× and 50× unlabeled probes. Arrow indicates shifted bands. (d) Transient expression of the *35S:TCP4* effector construct with the *Pro* _*VND*_ _*7*_ *:LUC* reporter construct in *N. benthamiana* leaves. Quantitative analyses of luminescence intensity are shown in (e). The *35S:LUC* construct was used as the positive control. The luminescence intensity of *Pro* _*VND*_ _*7*_ *:LUC* is set to 1. Relative luminescence intensity was calculated by normalizing to the value of *Pro* _*VND*_ _*7*_ *:LUC*. The data are the means ± SD of triplicate experiments. (*P < 0.01).

Our data showed that the activation of *TCP4* is responsible for secondary cell wall biosynthesis. To characterize this process in more detail, we searched for TCP4‐targeted genes among lignification‐related genes using the TCP‐binding motif ‘GTGGTCCC’ (Schommer *et al*., 2008) as bait. Intriguingly, we found one ‘GTGGTCCC’ *cis* element in the promoter region of *VND7* (Figure 4a). *VND7* is involved in xylem vessel formation (Yamaguchi *et al*., 2008). We first measured the levels of *VND7* transcripts in the different lines. As shown in Figure 4b, the *VND7* transcriptional level was lowest in the *jaw*‐D plants and up‐regulated in the *rTCP4‐OX* lines. An electrophoretic mobility shift assay (EMSA) revealed that the motif in *VND7* was bound by His‐TCP4 but not His alone. The additional unlabeled probes competed for binding in dose‐dependent manner (Figure 4c). The miR319‐resistant *rTCP4* cannot be sufficiently cleaved by miR319, due to a mutation at the cleavage site in *TCP4* (Schommer *et al*., 2008). We also found that rTCP4‐HIS bounds to the promoter region of the *VND7* (Figure 4c). The qRT‐PCR and EMSA analyses indicated that TCP4 might activate the transcription of *VND7* by directly binding to its promoter. Thus, the transient expression system was used next to investigate whether TCP4 activates the expression of *VND7*. When we co‐transfected the *ProVND7:LUC* reporter plasmid with the *35S:TCP4* effector plasmid, strong LUC activity was detected (Figure 4d,e). However, in the absence of the effector *35S:TCP4* plasmid, LUC activity was much lower (Figure 4d,e). These results revealed that TCP4 indeed activates transcriptional activity of *VND7 in vivo*.

**Figure 5:**
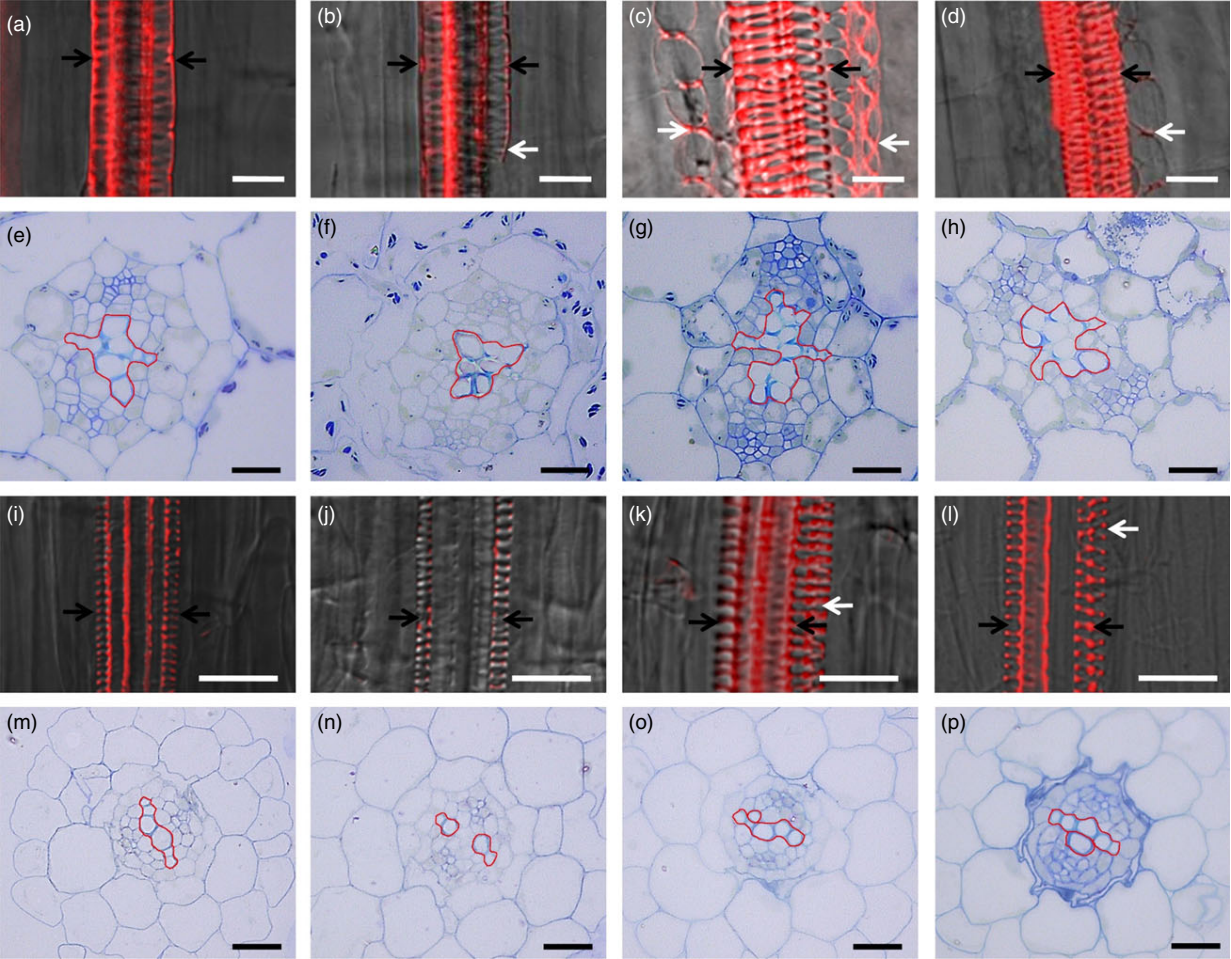
Modulation of vessel elements formation by miR319 and *TCP4*. Xylem vessels were stained with basic fuchsin red and visualized under a confocal microscope in (a–d) and (i–l). (a–d) Confocal laser scanning microscopy images of hypocotyls from Col‐0 (a), *jaw‐*D plants (b) and *rTCP4*‐*OX* lines (c and d). (e–h) Cross‐section of hypocotyls in the 1‐week‐old seedlings. (i–l) Confocal laser scanning microscopy images of roots from Col‐0 (i), *jaw*‐D plants (j) and *rTCP4*‐*OX* lines (k and l). (m–p) Cross‐section of roots in the 1‐week‐old seedlings. White and black arrows indicate protoxylem vessels. White arrows in (b) indicate the absence of vessels on one side. The phenotype was also found in (f). White arrows in (c, d) and (k, l) indicate the additional formation of vessels in the roots and hypocotyls, respectively. The phenotype was also found in (g, h) and (o, p). Bars = 20 μm.

Because *VND7* plays a pivotal role in regulating the differentiation of all types of xylem vessels in *Arabidopsis* (Yamaguchi *et al*., 2008), we then assessed vessel formation using specific basic fuchsin red staining of xylem and semi‐thin section. As shown in Figure 5, *jaw*‐D plants showed repressed vessel element differentiation (Figure 5b,f), but more vessel elements were observed in the hypocotyls of the *rTCP4*‐*OX* lines (Figure 5c,d,g,h). Similarly, the roots of the *jaw*‐D plants exhibited weaker signals in vessels (Figure 5j) and repressed vessel element differentiation (Figure 5n). In contrast, the *rTCP4*‐*OX* lines showed increased formation of vessel elements (Figure 5k,I,o,p). Overexpression of *rTCP4* in *jaw‐*D plants rescued the vessel element defects of hypocotyls and roots (Figure S1). These results corroborate that TCP4 is involved in the differentiation of xylem vessel elements.

### Quantitative RT‐PCR analysis of genes involved in secondary wall formation

**Figure 6:**
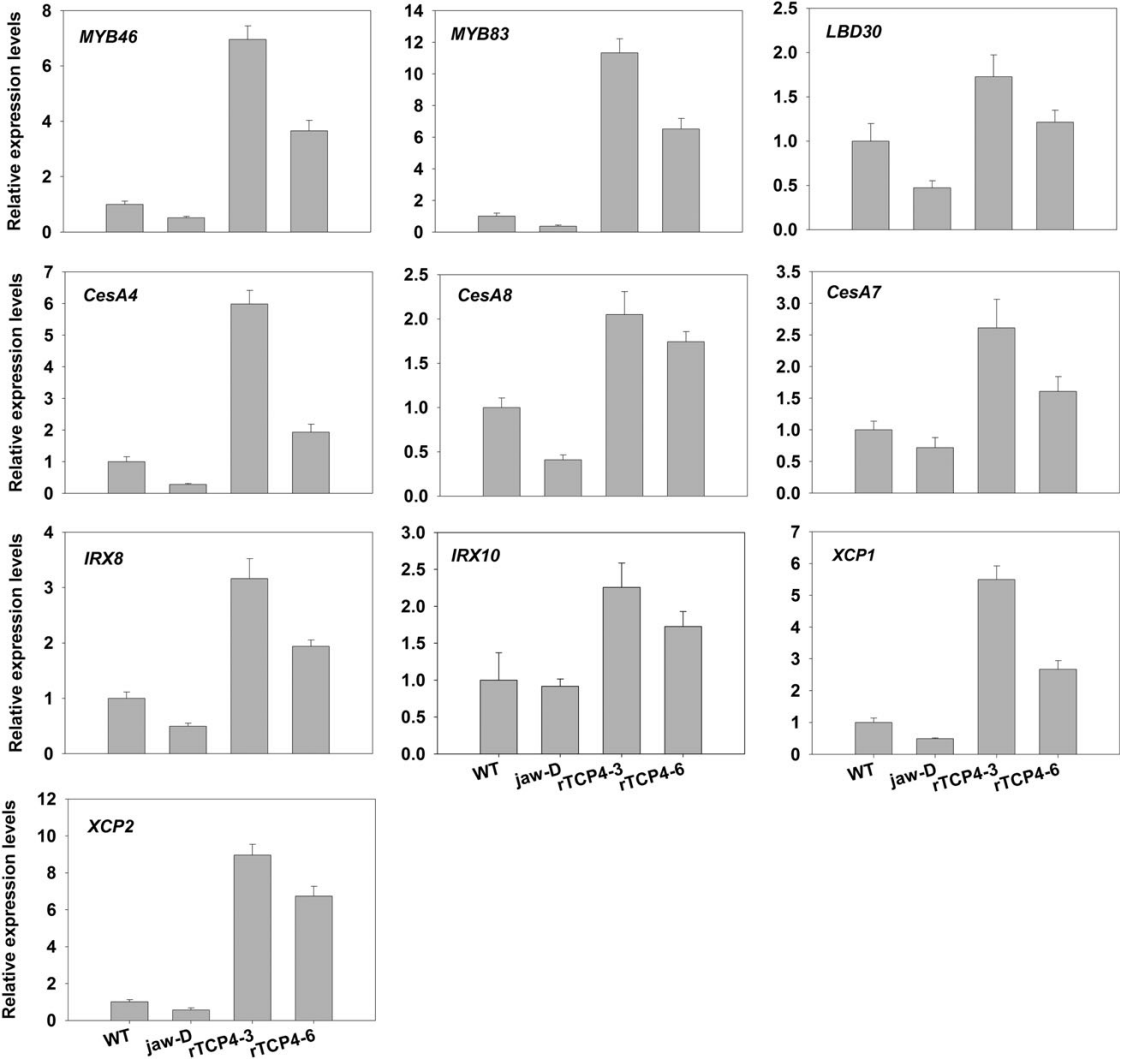
qRT‐PCR analysis of secondary wall‐associated formation genes in 2‐week‐old seedlings. Bars show the relative expression levels of each gene in the wild type, *jaw*‐D plants and *rTCP4*‐*OX* lines. The expression level of each gene in the wild type is set to 1. Relative gene expression was calculated by normalizing to the value in WT plants. Error bars represent SE of triplicate experiments.

Our data showed that TCP4 can bind the *VND7* promoter to modulate its transcriptional level. Previous studies showed that VND7 also modulates a series of transcription factor genes including *MYB46* and *MYB83*, which up‐regulate the expression of many genes related to secondary cell wall formation both in fibres and vessels (McCarthy *et al*., 2009; Zhong *et al*., 2007). *LBD30* forms a positive feedback loop with VND7 during xylem vessel formation (Soyano *et al*., 2008; Zhou *et al*., 2009). We found lower transcriptional levels of *MYBs* and *LBD30* in *jaw*‐D plants and higher transcriptional levels in the *rTCP4*‐*OX* lines compared with WT (Figure 6). CESA4/IRX5, CESA7/IRX3 and CESA8/IRX1 are cellulose synthases that function in the synthesis of the secondary cell wall (Taylor *et al*., 2003). *IRX8* and *IRX10* are required for normal amounts of hemicellulose and cellulose in secondary cell wall formation (Brown *et al*., 2005; Zeng *et al*., 2016). Here, we also found lower transcriptional levels of these genes in *jaw*‐D plants and up‐regulation in the *rTCP4*‐*OX* lines (Figure 6). Two plant cysteine proteases, *XCP1* and *XCP2*, that are also the direct target genes of VND7 (Yamaguchi *et al*., 2011), may be involved in autolysis of programmed cell death (PCD) during the xylem differentiation of tracheary elements (TEs) (Avci *et al*., 2008). The expression levels of these proteases strikingly corresponded to the *TCP4* transcript level.

**Figure 7:**
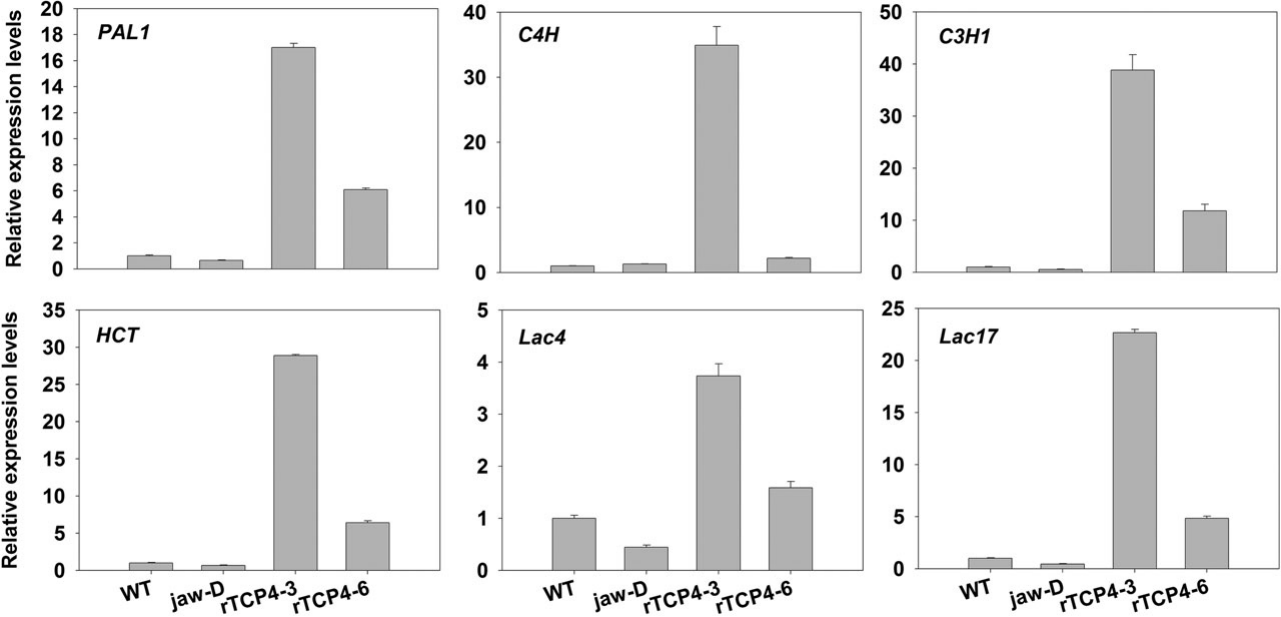
qRT‐PCR analysis of lignin biosynthesis genes in 2‐week‐old seedlings. Bars show the relative expression levels of each gene in the wild type, *jaw*‐D plants and *rTCP4*‐*OX* lines. The expression level of each gene in the wild type is set to 1. Relative gene expression was calculated by normalizing to the value in WT plants. Error bars represent SE of triplicate experiments.

Consistent with the histological data showing that *TCP4* overexpression caused ectopic deposition of lignin, we found that *TCP4* overexpression substantially induced the expression of genes involved in lignin biosynthetic pathways (Figure 7). Both laccase genes *LAC4* and *LAC17*, which are involved in secondary wall formation (Berthet *et al*., 2011; Zhou *et al*., 2009), were also activated by TCP4 (Figure 7).

**Figure 8:**
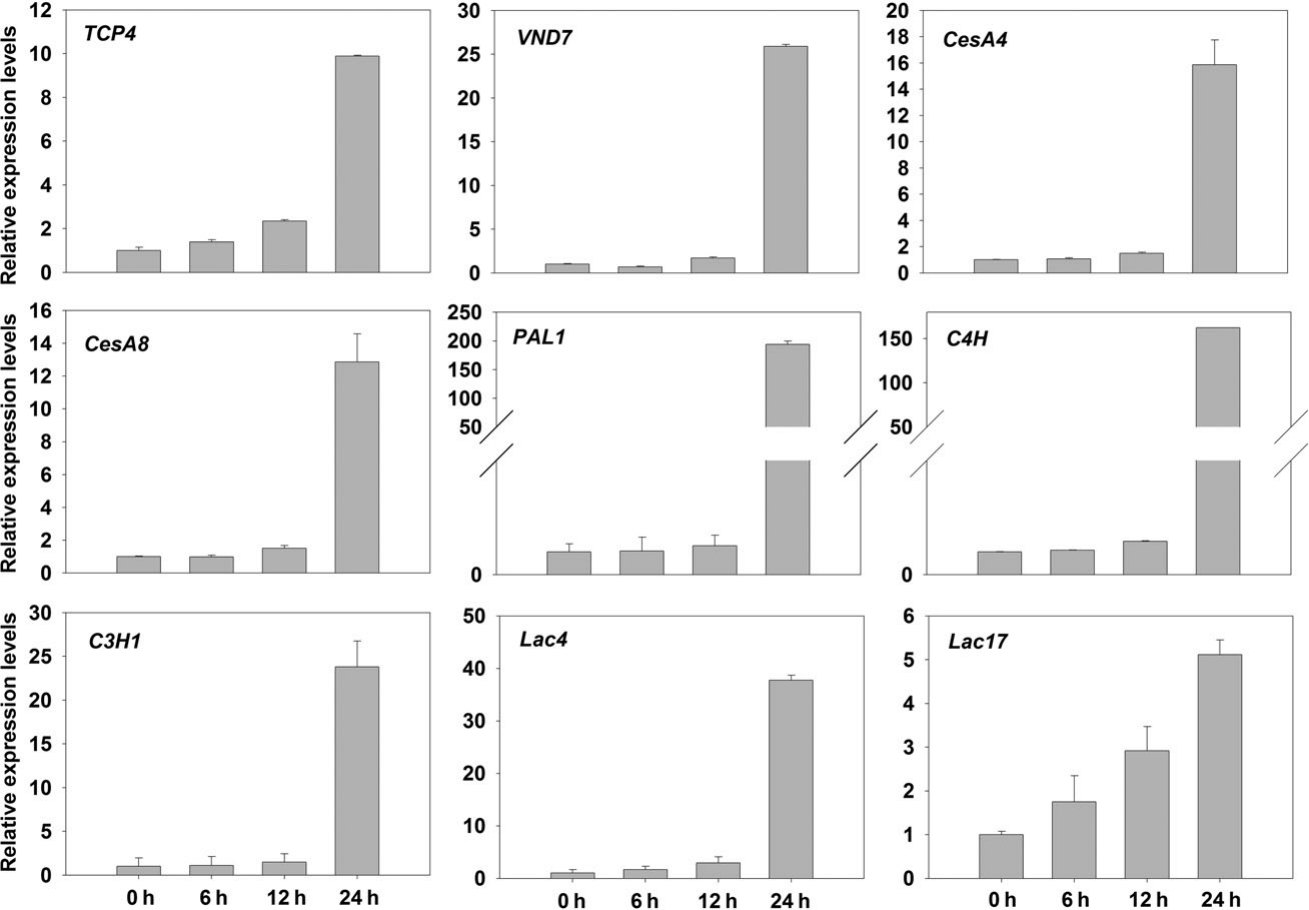
Induction of the secondary cell wall biosynthetic genes by estradiol activation of *rTCP4*. Bars show the relative expression levels of *TCP4*, *VND7* and secondary cell wall biosynthetic genes expression in 2‐week‐old *pER8‐rTCP4‐4* seedlings treated with 2 μm estradiol for various periods of time. The expression levels of *TCP4* and *VND7* in the 2‐week‐old *pER8‐rTCP4‐4* seedlings without treatment are set to 1, respectively. Relative gene expression was calculated by normalizing to the value in the 2‐week‐old *pER8‐rTCP4‐4* seedlings without treatment. Error bars represent SE of triplicate experiments.

To further confirm that TCP4 could activate the expression of cellulose and lignin biosynthesis genes, the *rTCP4* was subcloned into *pER8* (Zuo *et al*., 2000), downstream of an estrogen‐inducible promoter, and the resulting plasmids were introduced into wild‐type Arabidopsis via the floral‐dip method (Clough and Bent, 1998). Two‐week‐old transgenic plants were treated with 2 μm estradiol, and estradiol activation of *TCP4* substantially induced the expression of cellulose and lignin biosynthesis genes (Figure 8). Together, these results demonstrate that TCP4 is a transcriptional activator of cellulose and lignin biosynthesis during secondary wall formation.

### Inducible overexpression of *rTCP4* is sufficient to induce ectopic xylem vessel formation

**Figure 9:**
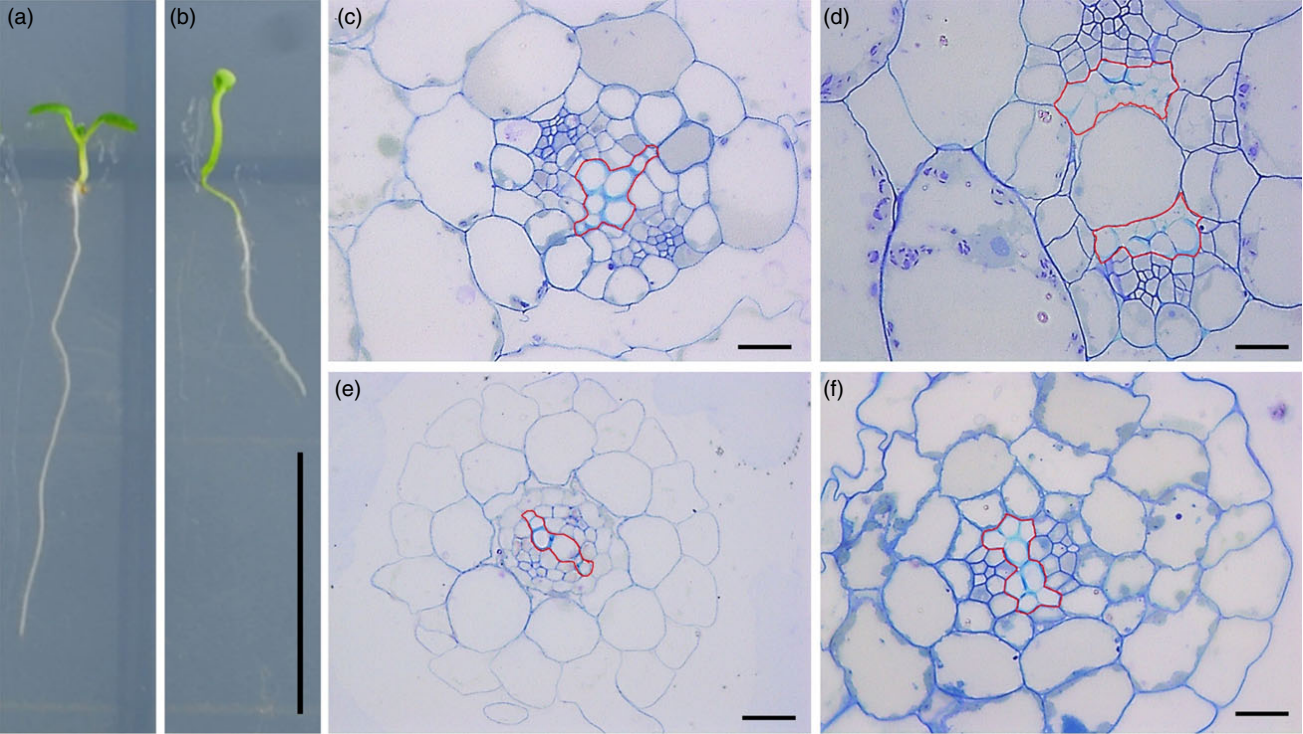
Inducible overexpression of *rTCP4* increased xylem vessel element formation in transgenic Arabidopsis. (a) Phenotype of 1‐week‐old *pER8‐rTCP4‐4* seedlings growing in MS medium without 2 μm estradiol. (b) Phenotype of 1‐week‐old *pER8‐rTCP4‐4* seedlings growing in MS medium with 2 μm estradiol. (c) Cross‐sections of hypocotyls in (a) showing similar phenotype with wild type. (d) Cross‐sections of hypocotyls in (b) showing increased xylem vessel formation. (e) Cross‐sections of roots in (a) showing similar phenotype with wild type. (f) Cross‐sections of roots in (b) showing increased xylem vessel formation. Bars = 1 cm (a, b), 20 μm (c–f).

To further confirm that *TCP4* is involved in xylem vessel formation, *pER8‐rTCP4‐4* transgenic seeds were grown in MS medium with or without 2 μm estradiol. Under estradiol‐free growth conditions, *pER8‐rTCP4‐4* seedlings showed a phenotype similar to the wild type (Figure 9a). In the presence of estradiol treatment, *pER8‐rTCP4‐4* seedlings exhibit long hypocotyls, epinastic cotyledons and smaller rosette leaves (Figure 9b). Cross‐sections of hypocotyls and roots of *pER8‐rTCP4‐4* seedlings without estradiol treatment did not show obvious differences from the wild type (Figure 9c,e). In contrast, estradiol treatment increased the production of xylem vessel elements in *pER8‐rTCP4‐4* seedlings (Figure 9d,f).

### Transcriptional levels of *TCP4* and *VND7* are regulated by developmental processes

**Figure 10:**
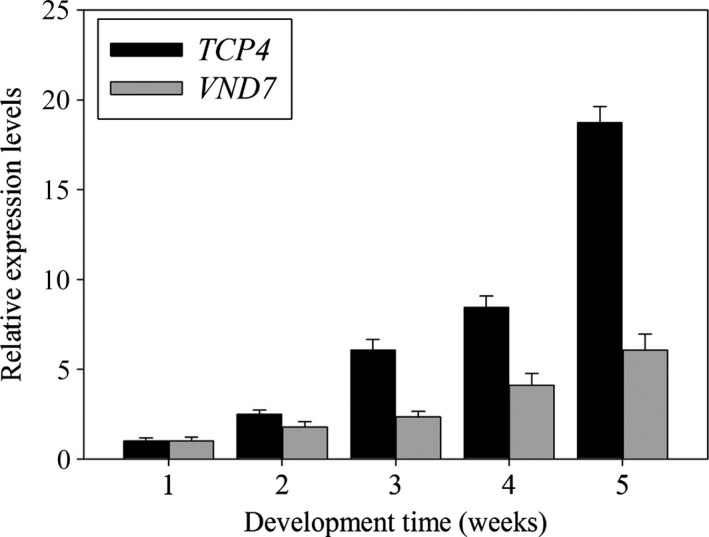
Dynamic expression of *Arabidopsis TCP4* and *VND7* mRNA. qRT‐PCR analysis of *TCP4* and *VND7* expression changes during development. The expression levels of *TCP4* and *VND7* in the 1‐week‐old wild‐type plants are set to 1, respectively. Relative gene expression was calculated by normalizing to the value in the 1‐week‐old wild‐type plants. Error bars represent SE of triplicate experiments.

The expression of *miR319* varied depending on the developmental state (Nag *et al*., 2009). As *TCP4* is the target gene of *miR319*, we measured the expression levels of *TCP4* and *VND7* during different developmental periods. As shown in Figure 10, we found that the transcriptional levels of *TCP4* and *VND7* gradually increased during plant development and reached a maximum level after 5 weeks of growth. This result indicates that a positive correlation exists between *TCP4* and *VND7*.

## Discussion

Previous studies demonstrated that miR319 and its targets, which comprise a set of TCP transcription factor genes, regulate various developmental physiological processes, such as leaf growth (Palatnik *et al*., 2003; Schommer *et al*., 2014), leaf senescence (Sarvepalli and Nath, 2011; Schommer *et al*., 2008) and petal development (Nag *et al*., 2009). Here, we discovered that miR319 and its target gene *TCP4* simultaneously control plant developmental processes and senescence by modulating *VND7*‐dependent xylem vessel formation and PCD. These findings suggest that plants strictly adjust *TCP4* levels to coordinate two key processes in plant biology: development and senescence.

Schommer *et al*. (2008) found that *jaw*‐D plants with high miR319 expression levels could delay senescence by reducing *TCP4* expression and jasmonic acid (JA) biosynthesis. Given that JA also mediates cell wall lignification (Denness *et al*., 2011), it is possible that the lower level of miR319 during maturity results in a high level of *TCP4* expression, which promotes JA synthesis, and that the produced JA synergistically contributes to lignin biosynthesis. Although exogenous application of JA caused premature senescence in attached and detached leaves in WT *Arabidopsis*,* TCP4*‐targeted *LOX2* expression was sharply reduced during leaf senescence (He *et al*., 2002). LOX2‐RNAi plants and WT plants did not show any differences in senescence initiation and progression based on the assessment of chlorophyll loss during natural senescence (Seltmann *et al*., 2010). These results indicate that the *TCP*‐targeted *LOX2* in the JA biosynthesis pathway is not necessary for promoting senescence in *Arabidopsis*, and a parallel pathway exists during the control of senescence. The differentiation of vessel cells results in an orchestrated construction of the secondary cell wall structure involving cellulosic thickening and lignification, PCD and cellular autolysis (Fukuda, 1997). Systematic analysis of gene expression revealed that many genes involved in both secondary wall formation and modification and PCD are simultaneously expressed just before morphological changes occur in TEs (Kubo *et al*., 2005; Ohashi‐Ito *et al*., 2010; Yamaguchi *et al*., 2010a, 2011). In this study, we showed that TCP4 binds to the promoter of *VND7* and activates its expression. VND7, an essential NAC transcription factor, activates downstream networks to promote PCD of TEs and xylem vessel differentiation (Yamaguchi *et al*., 2011). Overexpression of *VND7* causes seedlings to become pale in colour and results in their premature death (Yamaguchi *et al*., 2010a). TCP4 and VND7 showed a similar expression pattern in vascular tissue as shown by a promoter GUS assay (Koyama *et al*., 2007; Yamaguchi *et al*., 2008). During the promotion of maturation, the transcriptional levels of *TCP4* and *VND7* gradually increased during plant development. These findings suggest that *TCP4* initiates PCD, at least partly, by activating the *VND7* gene through the VND7 transcriptional network for secondary wall formation.

Development and senescence are widely studied processes that are fundamental for sessile plant survival in nature. Leaf senescence represents the final stage of leaf development and is critical for plant relocation of nutrients from the leaves to the reproducing seeds (Lim *et al*., 2007). Here, we uncovered a novel mechanism of miR319 in coordinating development and senescence in *Arabidopsis*. Development modulates miR319‐mediated *TCP4* expression to activate the downstream *VND7* network and thereby promotes secondary cell wall formation and PCD. Thus, low *TCP4* levels during the juvenile‐to‐adult/senescence transition retard xylem vessel differentiation. In contrast, high *TCP4* levels in adult/senescence plants promote rapid xylem vessel differentiation, which contributes to transport of water and minerals and provides mechanic strength to the entire plants, suggesting that proper levels of active *TCP4* are critical for plant development. Thus, this study reveals a novel role for miR319 in coordinating plant developmental processes with senescence responses.

## Experimental procedures

### Plant materials and growth conditions

*Arabidopsis thaliana* ecotype Columbia (Col‐0) was used as the WT control. *Jaw*‐D seeds were obtained from Prof. Detlef Weigel (Max Planck Institute for Developmental Biology). Surface‐sterilized seeds were germinated on Murashige and Skoog (MS) medium. The seeds were kept at 4 °C for 3 days and then transferred to a greenhouse (22 °C) under long‐day conditions (16 h light/day). Seedlings were transplanted to the soil 10 days after planting.

### Transgenic plants

The *ORF* of *rTCP4* was amplified with the primer pairs 5′‐ATGTCTGACGACCAATTCCATCACC‐3′ and 5′‐TCAATGGCGAGAAATAGAGGAAGCA‐3′ using *rTCP4‐GFP* plasmid (Schommer *et al*., 2008) and then used EZ cloning into *pBI121* and *pER8*, respectively. The resulting plasmids were introduced into *Agrobacterium* GV3101. Transgenic *Arabidopsis* lines expressing *rTCP4* were obtained by *Agrobacterium*‐mediated flower dipping transformation. The transformants were selected on MS medium containing 50 mg/L kanamycin for *pBI121*‐*rTCP4* and 20 mg/L hygromycin for *pER8‐rTCP4*.

### Histology

Inflorescence stems were collected at developmental stage 6.2 (Boyes *et al*., 2001), corresponding to a height of 15 cm. The basal parts of the stem were fixed at 4 °C overnight with 2% glutaraldehyde in PBS (33 mm Na_2_HPO_4_ and 1.8 mm NaH_2_PO_4_, pH 7.2). After fixation, tissues embedded in low viscosity (Spurr's) resin (Electron Microscopy Sciences, PA) were sectioned (1 μm thick) and stained with toluidine blue for light microscopy. For observation of subcellular structures, 85‐nm‐thick sections were poststained with uranyl acetate and lead citrate and observed using a JEM‐1230 transmission electron microscope (JEOL, Tokyo, Japan). The wall thickness of xylem vessel elements in the stem was measured.

For examination of lignified cell walls in stems, 50‐μm‐thick sections were stained for 5 min with 1% phloroglucinol in 6 N HCl to identify lignin, which was shown as a bright red colour. Semithin sections were processed according to Sun *et al*. (2013). One‐micrometer‐thick sections were stained for cellulose with 0.01% Calcofluor White and observed with an ultraviolet fluorescence microscope as described previously (Zhou *et al*., 2009). Under the conditions used, only secondary walls exhibited brilliant fluorescence. For vessel visualizations, the roots and hypocotyls of 7‐day‐old seedlings were cleared and stained with 0.01% basic fuchsin as described previously (Dharmawardhana *et al*., 1992).

### Protein expression and purification

The *TCP4* and *rTCP4* expression constructs *pET32a*‐*TCP4* and *pET32a*‐*rTCP4* were transformed into the *Escherichia coli* strain *Rosseta*™, respectively. LB (100 mL) containing 100 mg/mL ampicillin was inoculated with 1 mL of overnight culture and grown at 37 °C to mid‐log phase. Recombinant protein expression was induced with 0.5 mm isopropyl b‐L‐thiogalactoside. Cells were harvested after 3 h of induction. Cells were lysed according to the instructions of the MagneHis™ Protein Purification System (Promega, WI). The lysate was centrifuged, and the supernatant was loaded onto a Ni‐NTA spin column (Promega). Recombinant protein was eluted in a 200 μL volume containing 500 mm imidazole. The eluted protein was dialysed against 50 mm NaH_2_PO_4_, 300 mm NaCl and 10% glycerol for 6 h. The purification was monitored by Western blot using anti‐His HRP‐conjugated antibodies (Qiagen, Beijing, China).

### Emsa

A DNA probe was generated by end‐labelling a double‐stranded oligo (5′‐ATTGTTGGGTGGTCCCATAAAAAT‐3′) containing one TCP4‐binding site with a biotin label at the 3′ end. The binding reaction was conducted in a total volume of 20 μL containing 100 fmol of probe, 1× binding buffer (20 mm HEPES‐KOH, pH 7.8, 100 mm KCl, 1 mm EDTA, 0.1% BSA, 10 ng herring sperm DNA and 10% glycerol) and 1 μg of purified protein. The mixture was incubated for 30 min at room temperature and loaded on a 6% native polyacrylamide gel. Electrophoresis was conducted at 6 V/cm for 45 min with 0.25× Tris‐borate buffer at room temperature.

### Transactivation of *VND7* promoter activity by TCP4 in *Nicotiana benthamiana* leaves

The transient expression assays were performed in *N. benthamiana* leaves according to previously described methods (Chen *et al*., 2011). The *VND7* promoter was amplified with the primer pairs 5′‐TTTCATCAGTACCTGATCCAGC‐3′ and 5′‐GTGTCTTTTTGGAAGCTATTGC‐3′, cloned into the pMD18T vector (Takara, Dalian, China) and verified by sequencing. The *VND7* promoter was then fused with the luciferase reporter gene *LUC* through EZ cloning into the plant binary vector *pRI101* to generate the reporter construct *Pro*_*VND7*_ *:LUC*. The *TCP4* effector construct was *35S:TCP4*, and the *TCP4* coding region was amplified by PCR with the primer pairs. Five independent determinations were assessed. The experiment was repeated three times with similar results.

### Quantitative RT‐PCR

The aforementioned *Arabidopsis* seedlings were used for quantitative real‐time PCR (qRT‐PCR) analysis of gene expression. Total RNAs were extracted using Trizol reagent (Invitrogen, CA) and reverse transcribed using M‐MLV (Promega). Real‐time PCR was performed with the ABI7500 real‐time PCR system using *TransStart* ^®^ Top Green qPCR SuperMix (TransGen, Beijing, China). The relative gene expression level was calculated by normalizing against the internal control *Ubiquitin10*. Three technical replicates were carried out for each sample. Primers are listed in Table S1.

### Statistical analysis

Statistical analysis and exponential curve fitting were performed using SigmaPlot 10.0 (Systat Software Inc., San Jose, CA) software. Results are expressed as mean ± SD. Student's *t*‐tests were performed, and *P*‐values provided in results.

## Conflict of interest

The authors declare no competing interests.

## Supporting information


**Figure S1** Cross‐sections of hypocotyls and roots of F1 seedlings.

**Table S1** Primers used for qRT‐PCR analysis in this study.
