# Supplementary figures and images for: Activation of secondary cell wall biosynthesis by miR319‐targeted TCP4 transcription factor
Source: Plant Biotechnol J. 2017 Apr 27;15(10):1284–94. doi: 10.1111/pbi.12715 (PMC5595714; doi:10.1111/pbi.12715)

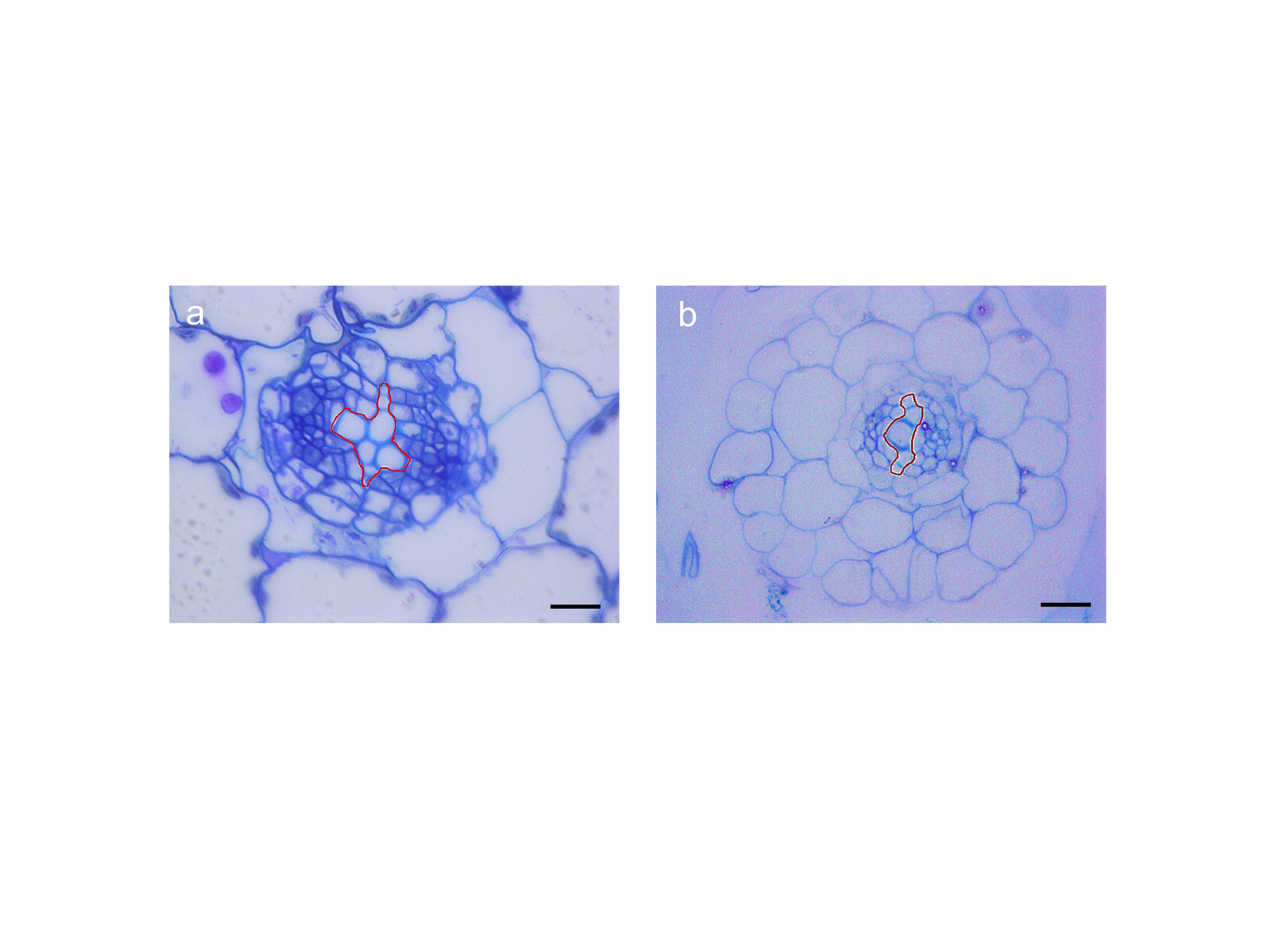

Supplement: Supplementary file 1 — Figure S1 Cross‐sections of hypocotyls and roots of F1 seedlings. [file PBI-15-1284-s001.jpg]
