# Supplementary material for: Activation of secondary cell wall biosynthesis by miR319‐targeted TCP4 transcription factor
Source: Plant Biotechnol J. 2017 Apr 27;15(10):1284–94. doi: 10.1111/pbi.12715 (PMC5595714; doi:10.1111/pbi.12715)
Supplement: Supplementary file 2 — Table S1 Primers used for qRT‐PCR analysis in this study. [file PBI-15-1284-s002.docx]

| Ubq10 qF | AACTTTGGTGGTTTGTGTTTTGG |
| --- | --- |
| Ubq10 qR | TCGACTTGTCATTAGAAAGAAAGAGATAA |
| TCP4 qF | ATACCAGCACGGTTTCAGGG |
| TCP4 qR | GCATGGAAGCTAGACAAGCC |
| VND7 qF | GGTAGAGACAAGGCGGTACT |
| VND7 qR | AGCTCGGAGTTTTGGAGACG |
| LBD30 qF | CTATCTACGGCTGCGTCTCTCACATCGT |
| LBD30 qR | TAGAGATCCTGAAGATGACACCGGAAC |
| MYB46 qF | GAATGTGAAGAAGGTGATTGGTACA |
| MYB46 qR | CGAAGGAACCTCAGTGTTCATCA |
| MYB83 qF | AACGTGGATCCTTCTCTCCTC |
| MYB83 qR | AGCCGAGTAGCTATTTGAGACC |
| CesA4 qF | GAGTGATGATAAAACGATGAGCAG |
| CesA4 qR | TCTCAAAATTCTTCTGCGACATTA |
| CesA7 qF | ATGGGTAGACAGAACAGAACACCAA |
| CesA7 qR | CTTCAGCAGTTGATGCCACACTT |
| CesA8 qF | CACTTCTTTGCCTCTTGTTGCTTAC |
| CesA8 qR | GAAGCTCGAGGACACTCGTTAAGAT |
| IRX8 qF | TCAAGAGCTGTCACATTAGAGCAT |
| IRX8 qR | ATGATCCGGTAGAGAAGTGAAAAC |
| IRX10 qF | GTGAGAAGGCACTGAACTGGACT |
| IRX10 qR | GACTTCTAATGTTTTTGAAGTGCT |
| XCP1 qF | TTGACCCATGAAGAGTTCAAAGGAAGA |
| XCP1 qR | GAAAGCGAACTCAGATTCCCTGTTG |
| XCP2 qF | TTGCGAGATGCAAAAGGAT |
| XCP2 qR | GCCAATGCCTTCAAGAGACT |
| PAL1 qF | ACACTGTCTCTCAAGTGGCG |
| PAL1 qR | ACGTTGCGCTACAAGGATCA |
| C4H qF | TCGACACAGTTCTTGGACCG |
| C4H qR | TGGAGGTTCATGTGAGGCAC |
| Lac4 qF | CAATCGGAGTTCCATCCGGT |
| Lac4 qR | CACGTCGTGTGTACCTCCAA |
| Lac17 qF | TAAGCTACGCAGCCTGAACA |
| Lac17 qR | GATTGCACGGGTTTGTTCCC |
| HCT qF | TTGATCACTCCGCTGGCATT |
| HCT qR | ACCAGAGAAACCATCTGCCG |
| C3H1 qF | GGGATATGATCACGGCAGGG |
| C3H1 qR | ATCCGGTCAAGTCCAACCAC |
